# Clear Cell Renal Cell Carcinoma with a Ureteral Thrombus

**DOI:** 10.1089/cren.2018.0067

**Published:** 2018-10-01

**Authors:** Jeff John, Alessandro P. Aldera, Sunil Sinha, John Lazarus

**Affiliations:** ^1^ Division of Urology, Department of Surgery, Groote Schuur Hospital, University of Cape Town, Cape Town, South Africa.; ^2^ Department of Urology, Frere Hospital and Walter Sisulu University, East London, South Africa.; ^3^ Division of Anatomical Pathology, National Health Laboratory Service, University of Cape Town, Cape Town, South Africa.

**Keywords:** RCC, tumor, thrombus, ureter

## Abstract

***Introduction:*** Cancers of the kidney, arising from either the renal parenchymal tissue or the renal pelvis, is among the 13 commonest types of malignancy globally, accounting for between 3% and 4% of all newly diagnosed cancers. Renal cell carcinoma (RCC) accounts for 85% of all malignant renal neoplasms. We present a rare case of an RCC directly extending into the renal pelvicalyceal system and with a thrombus within the ureter.

***Case Presentation:*** A 39-year-old woman presented with a long-standing history of worsening left flank pain, intermittent visible hematuria, and a fullness in the left flank. Apart from an ill-defined left flank mass with pain on palpation, there was nothing remarkable on clinical examination. Contrast-enhanced abdominal CT scan images showed a large, heterogeneously enhancing soft tissue mass arising from the lower pole of the left kidney. The collecting system and the left proximal ureter were poorly visualized. A tentative diagnosis of RCC, cT_3a_N_0_M_0_ was made. Intraoperatively, we identified a large, left lower pole renal mass, displacing the pedicle superiorly. In addition, we found a bulky, dilated proximal ureter. A decision was made intraoperatively to proceed with radical nephrectomy and ureterectomy.

***Conclusion:*** We report a rare case of RCC directly invading from the renal pelvis and down the ureter as a thrombus mass, with no microscopic individual tumor implants in the ureter wall, invasion of the renal vein, or invasion of adjacent organs. To our knowledge, only four such cases have been reported in English literature, and as a result, very few theories explaining renal pelvic invasion and direct growth down the ureter have been postulated. This highlights the significance of adding invasion of the pelvicalyceal system as part of the most recent, updated tumor node metastases classification. In future, consideration needs to be given to include the extension of RCC into the ureter and/or bladder.

## Introduction

Cancers of the kidney, arising from either the renal parenchymal tissue or the renal pelvis, is among the 13 commonest types of malignancy globally, accounting for between 3% and 4% of all newly diagnosed cancers. Renal cell carcinoma (RCC) accounts for 85% of all malignant renal neoplasms. It is a highly aggressive tumor, making it the most lethal of all urological malignancies. With the widespread availability of cross-sectional abdominal imaging techniques, the absolute incidence of renal cancer shows an increasing trend over the last few years.

We present a rare case of an RCC directly extending into the renal pelvicalyceal system and with a thrombus within the ureter.

## Case Presentation

A 39-year-old, HIV-negative woman presented with a 1-year history of worsening left flank pain, intermittent visible hematuria, and a fullness in the left flank. In addition, she reported constitutional symptoms such as loss of appetite and significant loss of weight. She had previously been treated five times by her local general practitioner for recurrent urinary tract infections.

**FIG. 1.**
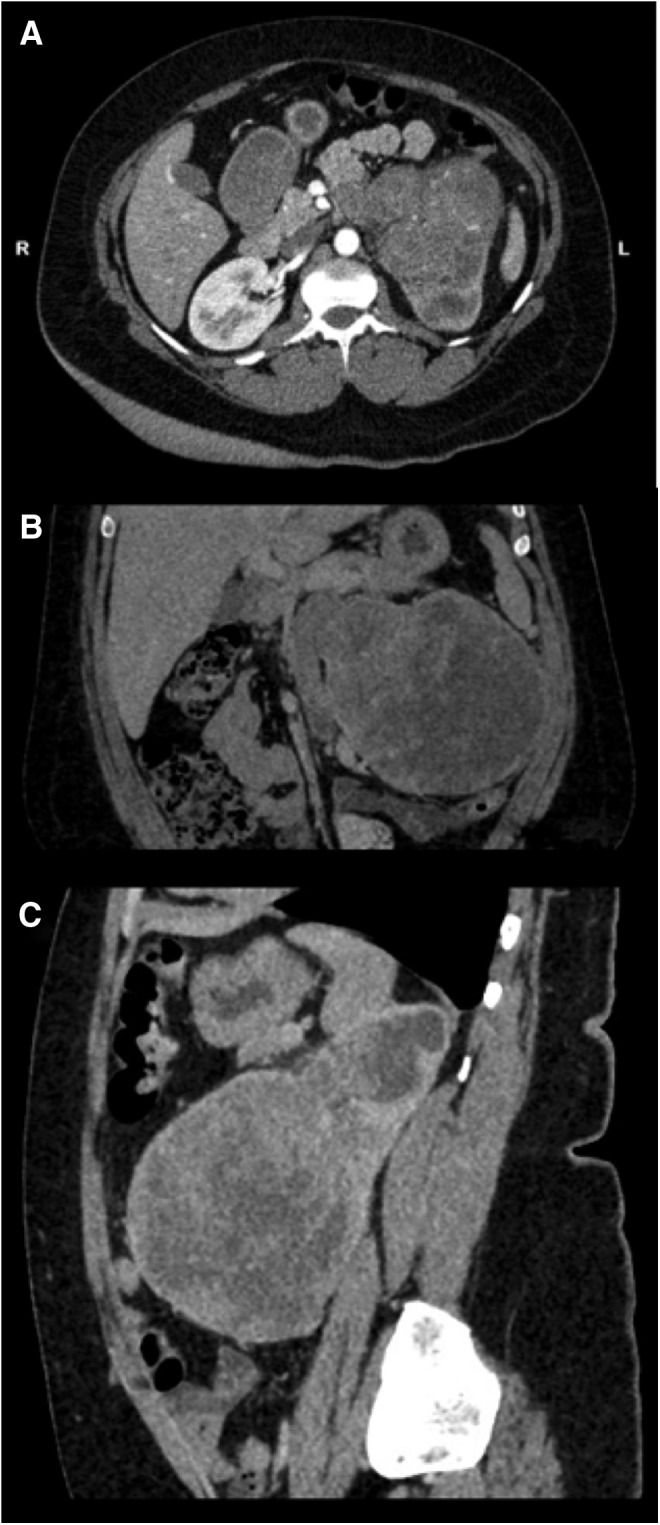
Axial **(A)**, coronal **(B)**, and sagittal **(C)** view of the contrast-enhanced abdominal CT scan showing a large, heterogeneously enhancing soft tissue mass arising from the lower pole of the left kidney.

The only relevant finding on clinical examination was that of an ill-defined left flank mass with pain on palpation. Apart from a normocytic, normochromic anemia, laboratory work was unremarkable. Contrast-enhanced abdominal CT scan images (Fig. 1) showed a large, heterogeneously enhancing soft tissue mass arising from the lower pole of the left kidney. It measured ∼108 × 106 × 105 mm (transverse, anterior-posterior, craniocaudal). The collecting system and the ureter were poorly visualized. The mass displaced the left renal artery superiorly and the left renal vein was not well demonstrated. The inferior vena cava appeared patent. There was no evidence of metastatic spread to the adrenal glands, intra-abdominal lymph nodes, liver, or chest. No bladder lesions were identified on cystoscopy.

A tentative diagnosis of RCC, cT_3a_N_0_M_0_ was made, and we planned for a hand-assisted laparoscopic radical nephrectomy. Intraoperatively, we identified a large, left lower pole renal mass displacing the pedicle superiorly. In addition, we found a bulky, dilated proximal ureter. A decision was made intraoperatively to proceed with radical nephrectomy as well as ureterectomy. The ureter was mobilized caudally and divided at the level of the pelvic brim. Since no enlargement of lymph nodes was found preoperatively on radiological imaging and no enlarged lymph nodes were detected when palpated intraoperatively, a lymph node dissection was not performed.

The patient had an uneventful postoperative course and was discharged 4 days postsurgery.

**FIG. 2.**
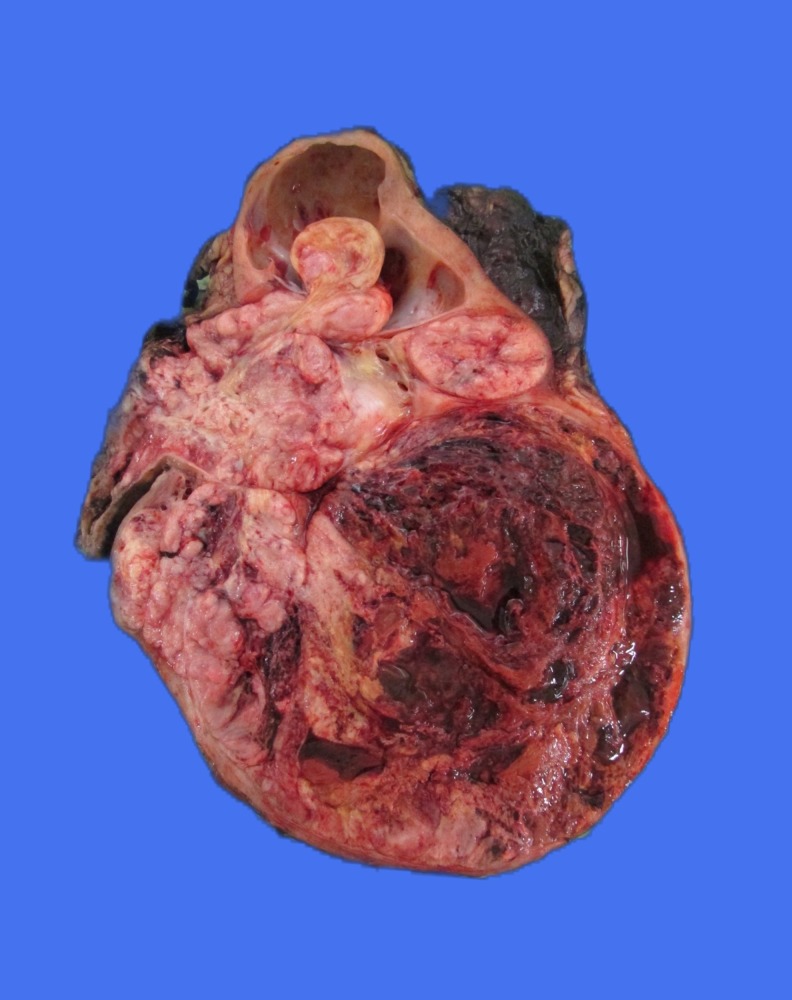
The cut surface of the bisected kidney showing a large, solid, unicentric, well-circumscribed tumor in the inferior pole of the left kidney with hemorrhage and necrosis.

**FIG. 3.**
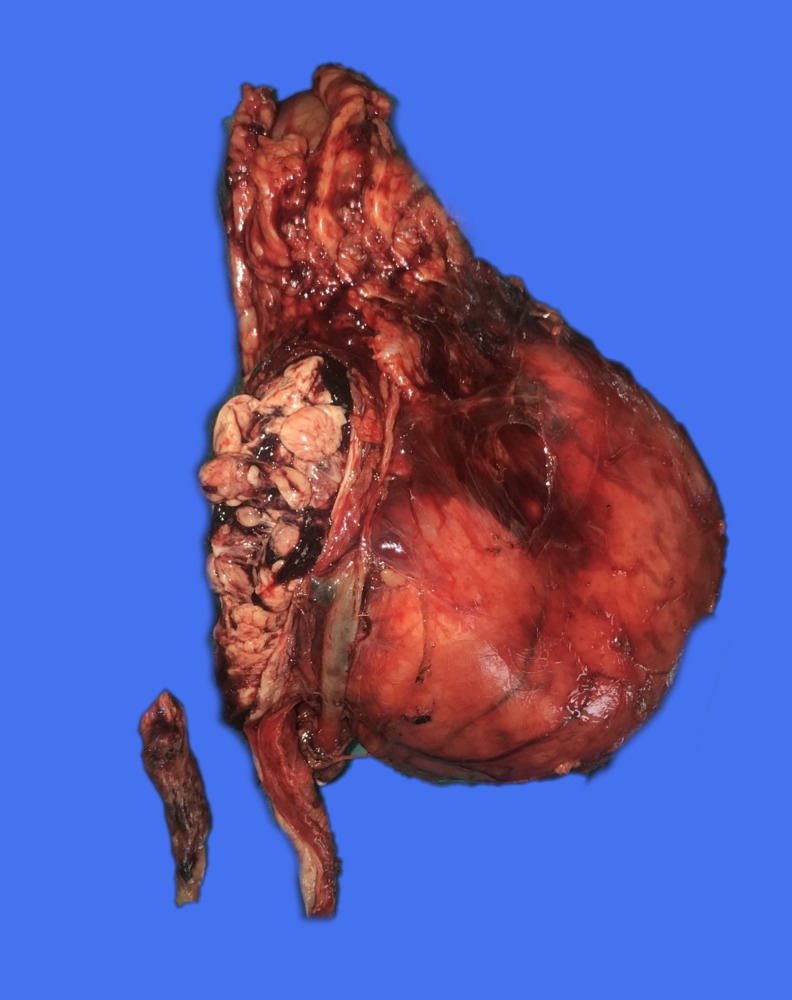
Extensive tumor involvement of the pelvicalyceal system with a tumor thrombus, seen here from within the opened ureter.

Macroscopically (Figs. 2 and 3), the cut surface of the bisected kidney showed a large, solid, unicentric, well-circumscribed tumor in the inferior pole of the left kidney with hemorrhage and necrosis. There was extensive involvement of the pelvicalyceal system and associated hydronephrosis. The renal sinus demonstrated the presence of a tumor thrombus within the opened ureter. The renal vein was uninvolved. The tumor capsule was intact, and Gerota's fascia was uninvolved. The margins, including the distal ureteric margin, were uninvolved by the tumor. The overall morphological features and immunophenotype were in keeping with an eosinophilic variant of clear cell RCC (ccRCC). The nuclear features corresponded with World Health Organisation/International Society of Urological Pathology grade 3. There was extensive coagulative tumor necrosis, but no evidence of sarcomatoid differentiation or lymphovascular invasion.

## Discussion

This case report is of a rare case of an RCC extending into the renal pelvicalyceal system and with a thrombus-like growth inside, but not invading the ureter wall.

RCC may extend into the major veins or perinephric tissues (pT3) or invade beyond Gerota's fascia (pT4). Venous invasion of the RCC is a poor prognostic sign. Often patients with venous involvement already have distant metastatic spread. In the absence of metastatic spread, the long-term survival rates are less than 60%. The first edition of the American Joint Committee of Cancer's (AJCC) tumor node metastases (TNM) staging system in 1978 included the involvement of the collecting system as part of the classification. In editions two to seven, invasion of the urinary collecting system was no longer a criterion. However, in the recent eighth edition, released in 2017, invasion of the pelvicalyceal system was added (pT3a) to the classification, and this is relevant in our case. Tumor involving the collecting system appears to be a poor prognostic factor in RCC patients. Hence, our patient needed to be followed up closely.

To our knowledge, only four such cases have been reported in the English literature. Gulati and colleagues in 2007 and Katukani and coworkers in 2013 presented cases of RCC invading the collecting system with direct extension down the ureter and protruding from the ureteral orifice in the bladder.^1,2^ In 2011, Fujita and colleagues reported a case of RCC with tumor thrombus formation in the renal pelvis and the whole ureter.^3^ On histopathological analysis, all three cases were reported as ccRCC. The most recent case reported by Parikesit and colleagues in 2016 was of a mixed type between ccRCC and papillary RCC type 1.^4^

Due to the rarity of such cases, very few theories explaining the renal pelvic invasion and direct growth down the ureter have been postulated. These include the dissemination of tumor cells by lymphatic spread or due to venous thrombus into the urinary tract.^1,3^ However, on microscopic analysis, there was no evidence of malignancy in the renal pelvic or ureteric mucosa, no renal vein involvement, and no lymphovascular invasion, and so it must have extended down the ureter by direct extension.

## Conclusion

We report a rare case of RCC directly invading from the renal pelvis and down the ureter as a thrombus mass, with no microscopic, individual tumor implants in the ureter wall, invasion of the renal vein, or invasion of adjacent organs. This further highlights the significance of adding invasion of the pelvicalyceal system as part of the recently updated AJCC TNM classification. In future, consideration needs to be given to include the extension of RCC into the ureter and/or bladder.

## Abbreviations Used

AJCC: American Joint Committee of Cancer
ccRCC: clear cell renal cell carcinoma
CT: computed tomography
RCC: renal cell carcinoma
TNM: tumor node metastases
